# Supplementary material for: Environmentally stable interface of layered oxide cathodes for sodium-ion batteries
Source: Nat Commun. 2017 Jul 26;8:135. doi: 10.1038/s41467-017-00157-8 (PMC5526914; doi:10.1038/s41467-017-00157-8)
Supplement: Supplementary file 1 — Supplementary Information [file 41467_2017_157_MOESM1_ESM.pdf]

File name: Supplementary Information

Description: Supplementary Figures and Tables.

File name: Peer Review File

Description:

SUPPLEMENTARY INFORMATION

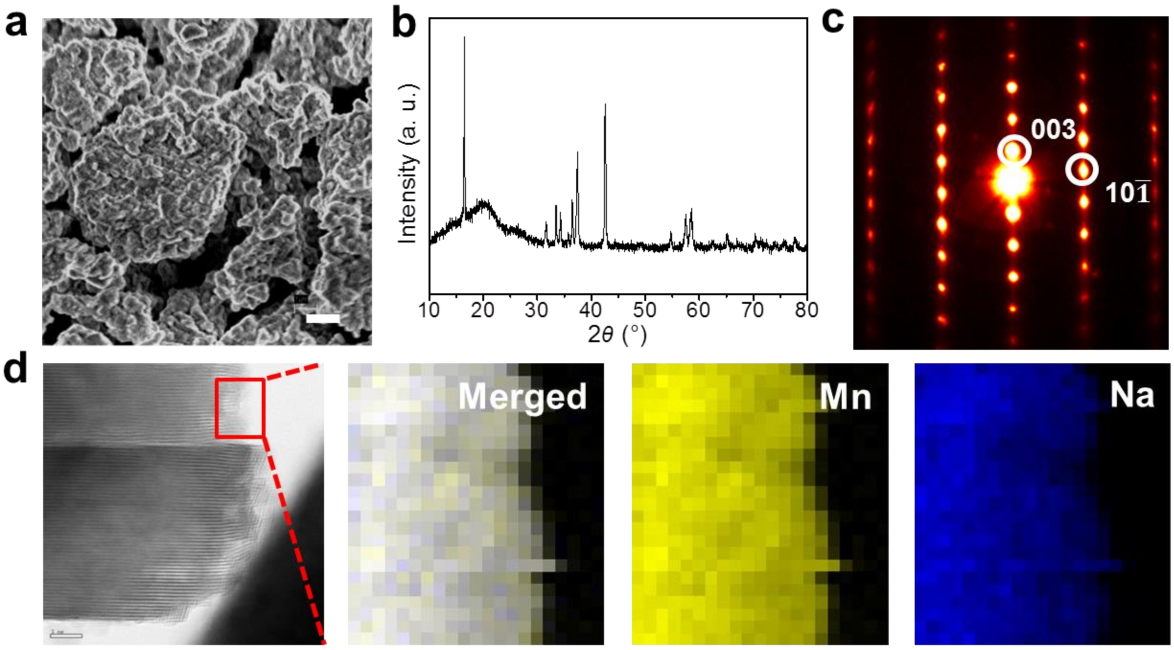

Supplementary Figure 1 | Bulk structures of the NM samples. (a) SEM image; scale bar, 1μm. **(b)** XRD plots; **(c)** ED patterns; **(d)** EELS mapping.

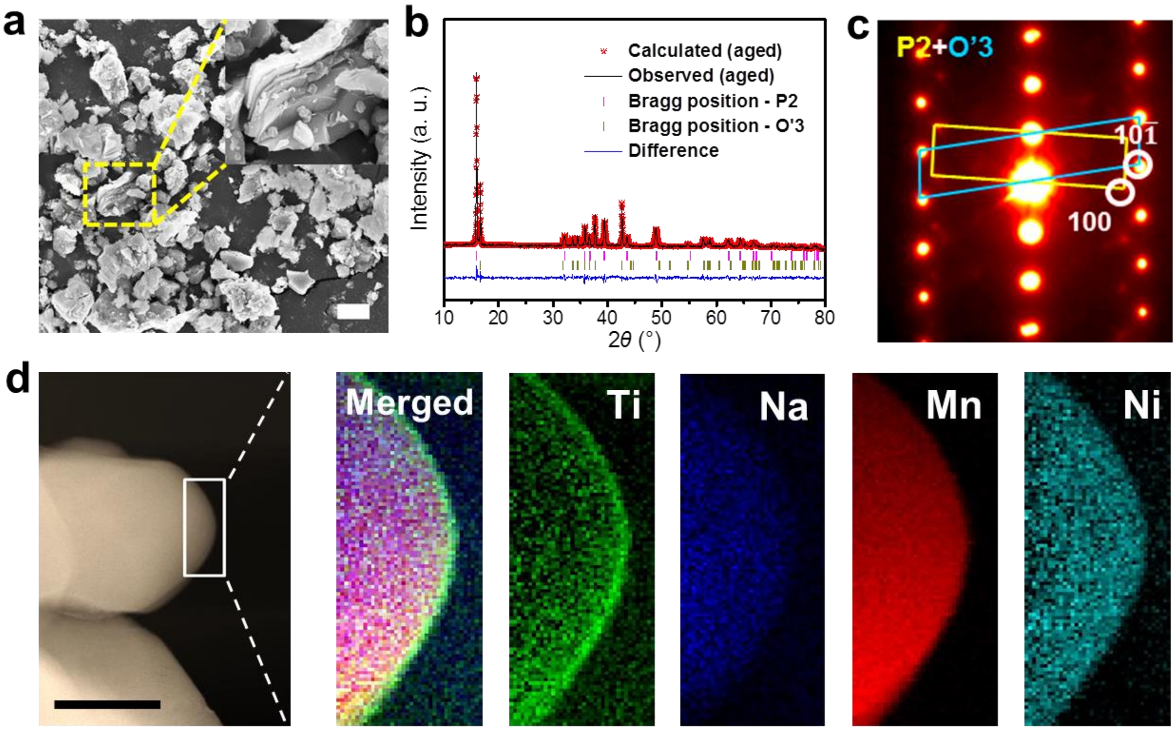

Supplementary Figure 2 | Bulk structures of the NMTN samples. (a) SEM image, inset shows the sheet-like morphology; scale bar, 2μm. **(b)** XRD and refinement plots with experimental data in black dots, calculated curves in red, difference curve in blue, and standard Bragg diffraction peaks in pink vertical bars; **(c)** ED patterns; **(d)** EELS mapping. Results indicate the apparent titanium enrichment in the interface; scale bar, 100nm.

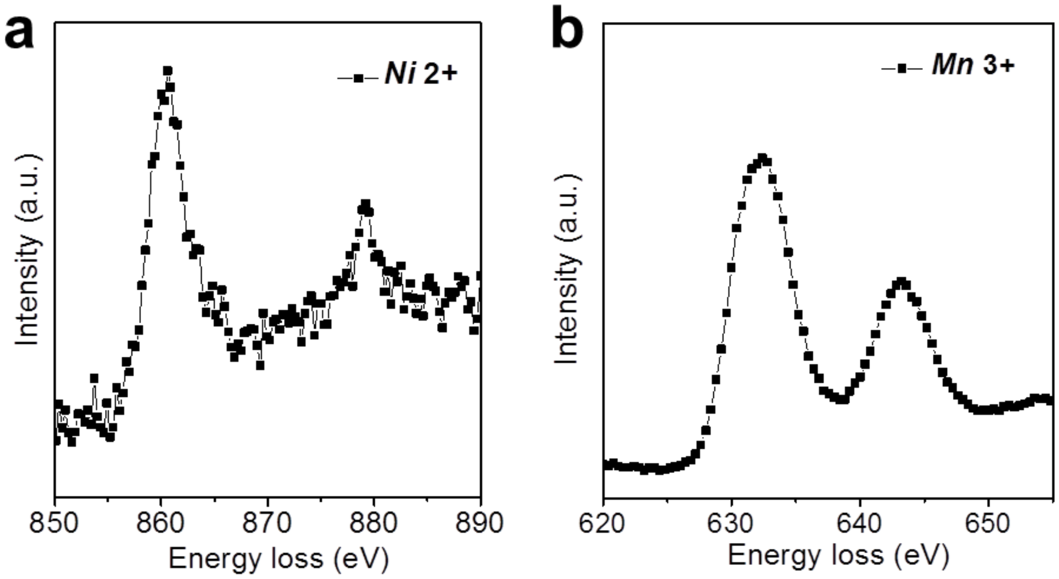

Supplementary Figure 3 | Valence states of the NMTN samples. EELS spectras of Ni-$L_{2,3}$ (a) and Mn$L_{2,3}$ (b) edges.

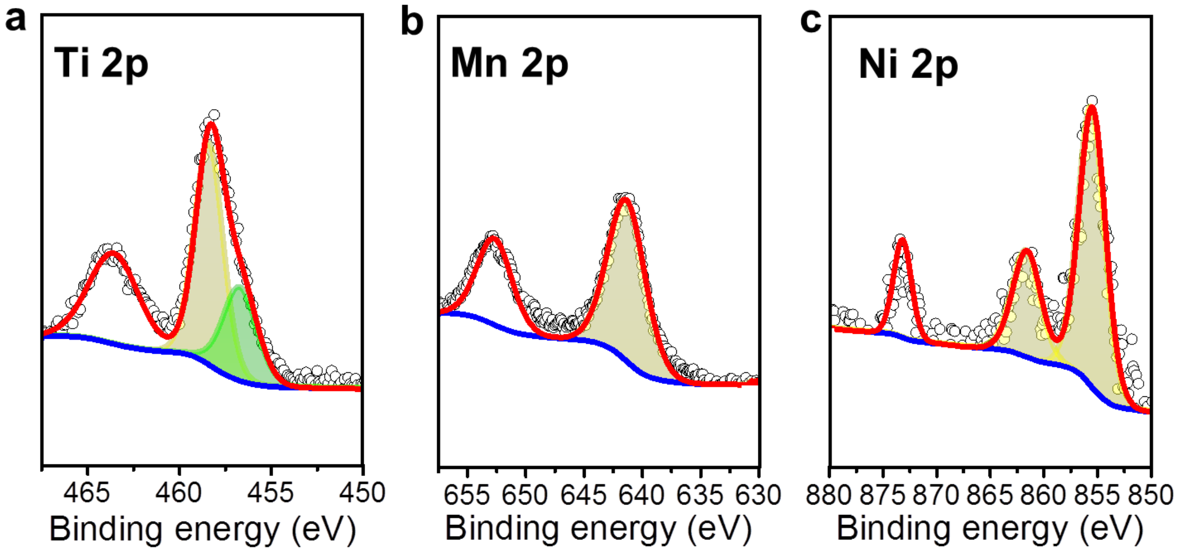

Supplementary Figure 4 | Valence states of the NMTN samples. XPS spectra of Ti 2p (a), Mn 2p (b), and Ni 2p (c) in NMTN samples.

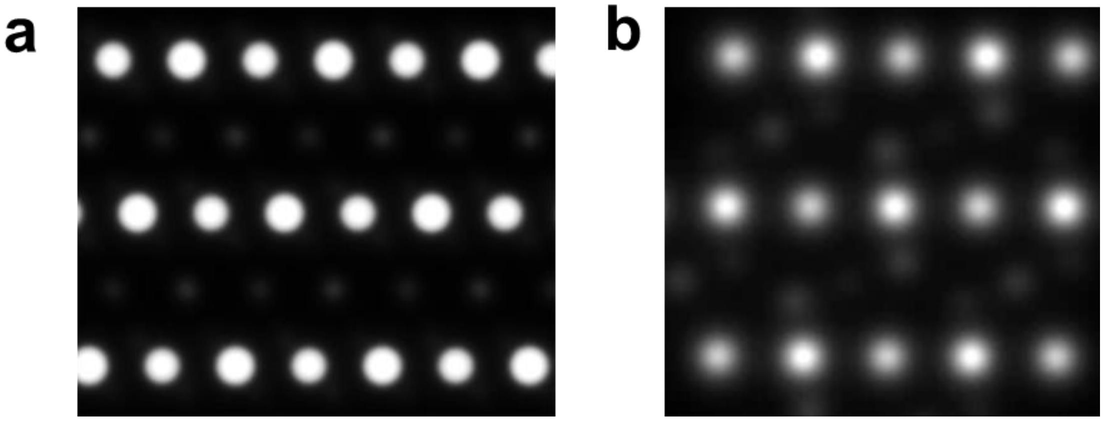

Supplementary Figure 5 | Simulated STEM results of the NMTN interface. Simulated HADDF-STEM images for layered O’3 (a) and spinel-like (b) structures. Simulated data is well agreeable with the experimental results.

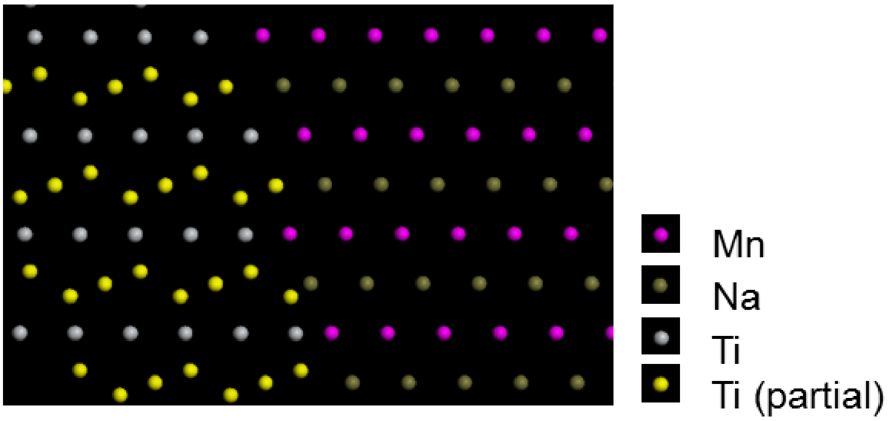

Supplementary Figure 6 | Simulated atomic-scale interface between layered O'3 and spinel-like structures. The brown, grey, white, and yellow balls represent the manganese, sodium, fully-occupied titanium, and partially-occupied titanium ions.

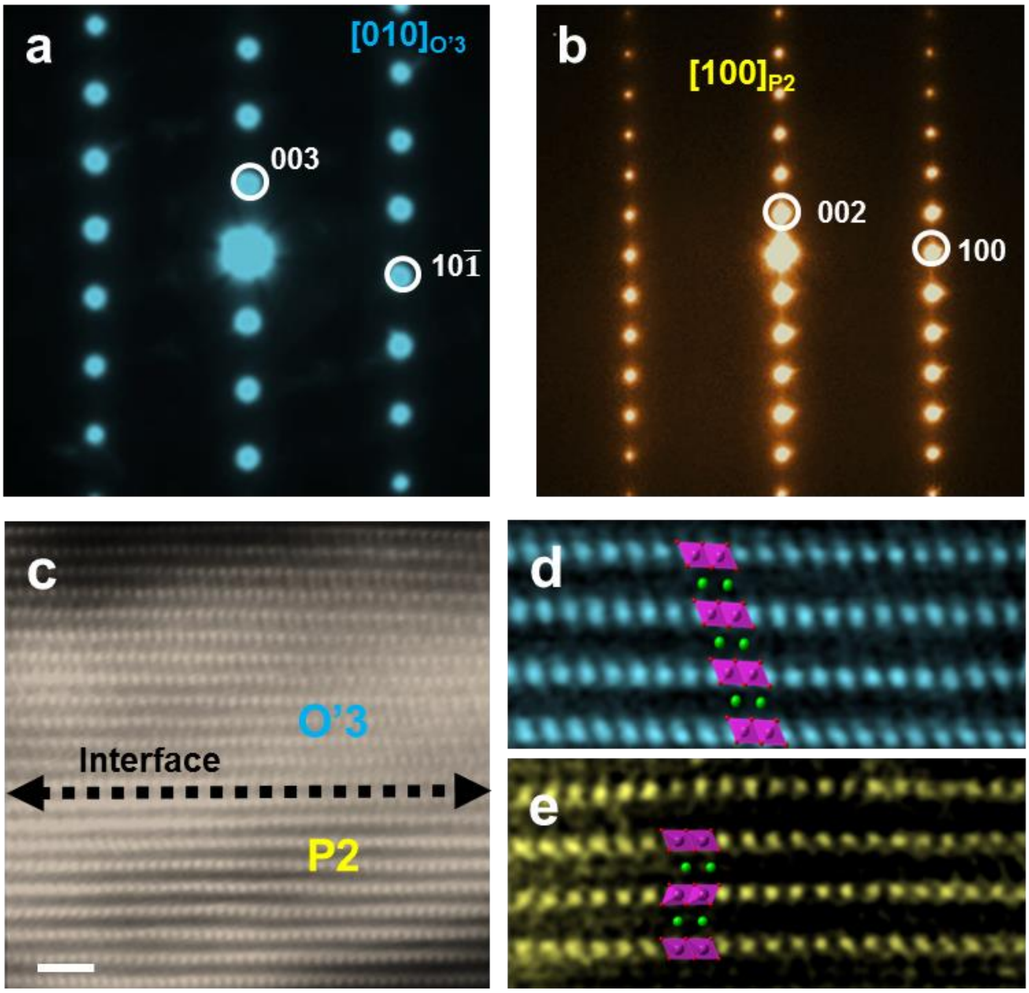

Supplementary Figure 7 | Atomic characterization of the bulk structure for the NM samples. The SAED patterns for momoclinic O’3 **(a)** and hexagonal P2 **(b)**; The HADDF image **(c)**, showing the grain boundary between O’3 and P2 phases. Zoom-in images are extracted from **(c)**, indicative of the different sequences of oxygen stacking for O’3 **(d)** and P2 **(e)**. Scale bar, 1 nm.

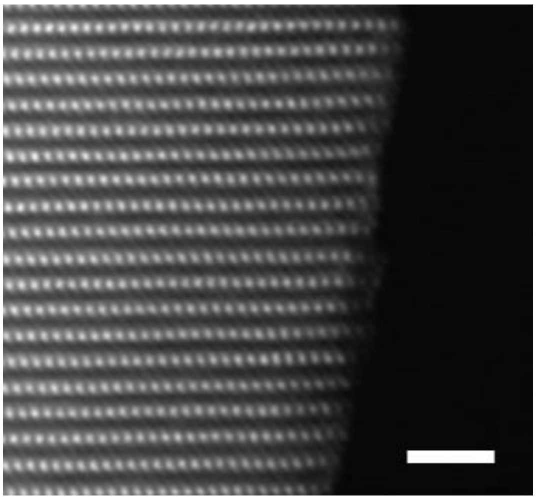

Supplementary Figure 8 | HADDF-STEM image of the NM samples; scale bar, 2nm.

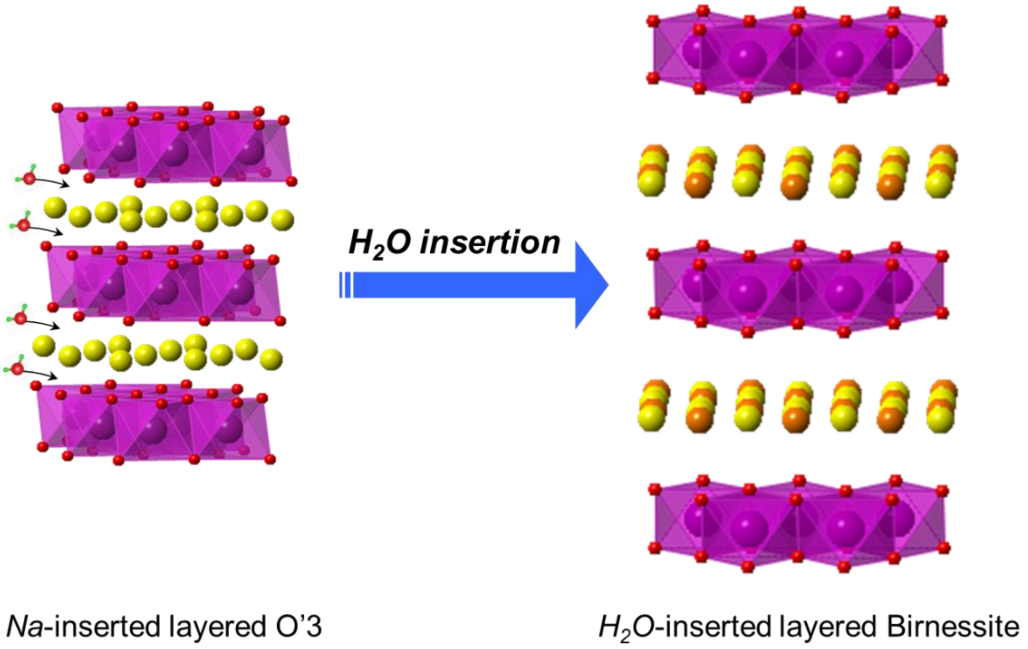

Supplementary Figure 9 | Schematic of water insertion into layered O'3 structure. Note that the lattice water is represented by brown balls in the right figure for simplicity.

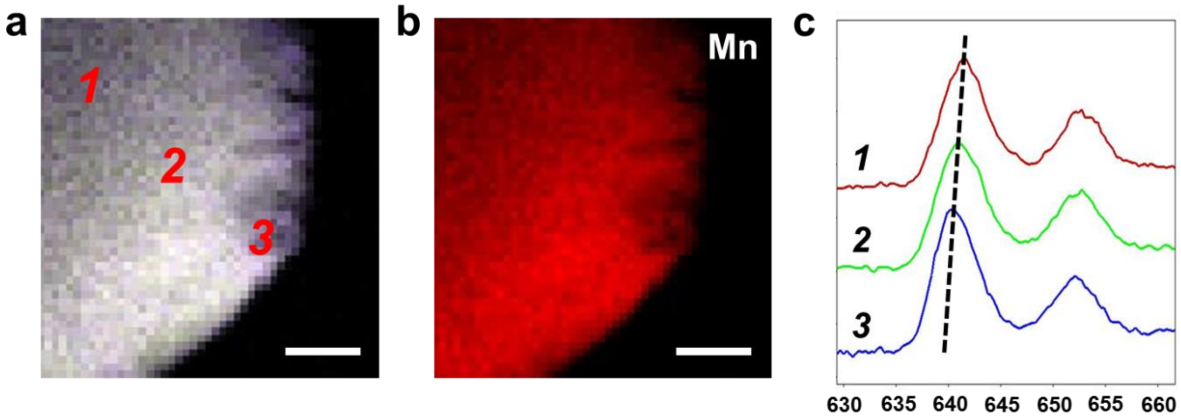

Supplementary Figure 10 | Characterization of the NM electrode after 20th charge. (a) The typical TEM image, the numerical marks indicate the selective spots for EELS spectras; Scale bar, 5nm. **(b)** EELS mapping of Mn element; Scale bar, 5nm. **(c)** corresponding EELS spectras, showing the gradual valence change from tetravalence (core) to bivalence (surface).

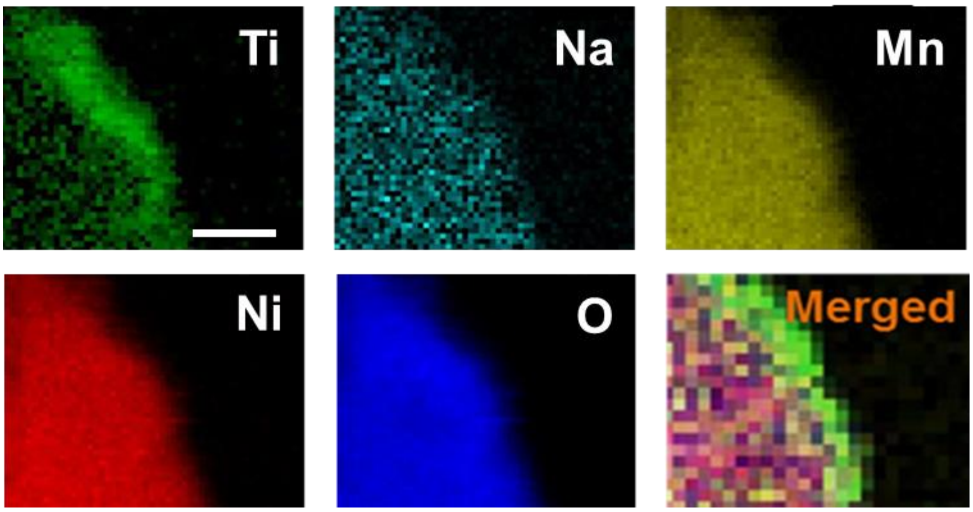

Supplementary Figure 11 | Characterization of the NMTN electrode after 20th charge. EELS mapping indicates the stable titanium-enriched interface is maintained during cycling. Scale bar, 5nm.

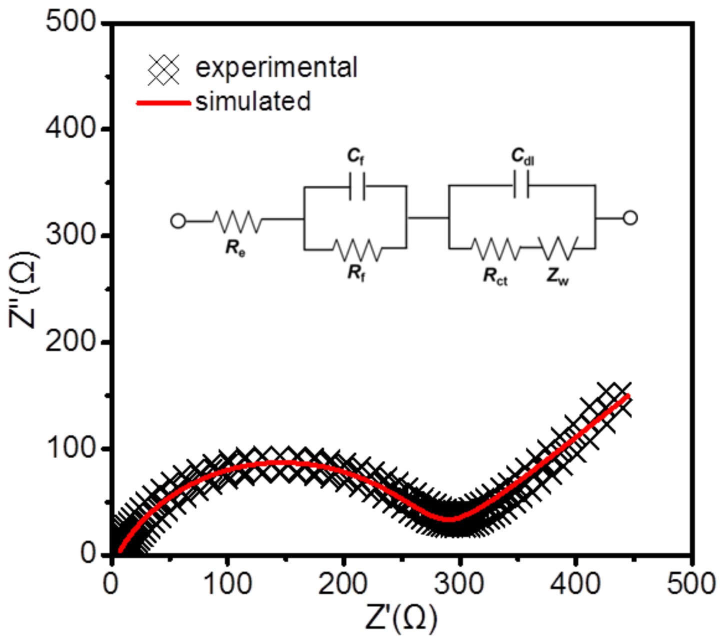

Supplementary Figure 12 | Impedance spectra and fitting pattern of the fresh NM electrode. The inset shows Randles equivalent circuit for simulation.

Supplementary table 1 | Refinement results of NMTN samples

Phase	P2		O’3	
Weight fraction (%)	59.5		40.5	
Space Group	P63/mmc		C2/m	
Cell parameters	a (Å)	2.8941(2)	a (Å)	5.6539 (5)
	b (Å)	2.8941(2)	b (Å)	2.8601 (2)
	c (Å)	11.1414(3)	c (Å)	5.7974 (3)
	α (°)	90.000 (0)	α (°)	90.000 (0)
	β (°)	90.000 (0)	β (°)	113.020 (5)
	γ (°)	120.000 (0)	γ (°)	90.000 (0)
	Volume (Å3)	80.818(8)	Volume (Å3)	86.282 (6)
Agreement factors	Rwp (%)		3.61	
	Rp (%)		2.78	
	$\chi 2$		1.649	

Supplementary table 2 | ICP results of Mn in Na anode surface

Samples	Mn (ppm)
Fresh cell (NM)	~0.00
100th cycled cell (NM)	~0.73
Fresh cell (NMTN)	~0.00
100th cycled cell (NMTN)	~0.00

Supplementary table 3 | Fitting results for impedance

Samples	R_f (Ω)	R_{ct} (Ω)
Fresh cell (NM)	21.4	259.1
20th cycled cell (NM)	39.5	322.7
100th cycle cell (NM)	128.3	411.8
Fresh cell (NMTN)	16.9	180.1
20th cycled cell (NMTN)	20.2	204.5
100th cycle cell (NMTN)	26.3	235.8

Supplementary table 4 | A comparison of some layered cathode materials

Cathode	Cycle life	Capacity	Rate
O'3/P2-NMTN (this work)	500 cycles (81%)	~186 mAh g^{-1}	10C (~114 mAh g^{-1})
O3-$NaNi_{1/2}Mn_{1/2}O_2$ ref. 24	50 cycles (75%)	~125 mAh g^{-1}	1C (~105 mAh g^{-1})
P2-$Na_{2/3}Ni_{1/3}Mn_{2/3}O_2$ ref. 50	50 cycles (95%)	~88 mAh g^{-1}	2C (~62 mAh g^{-1})
P2-$Na_{2/3}Ni_{1/3}Mn_{1/2}Ti_{1/6}O_2$ ref. 51	20 cycles (80%)	~127 mAh g^{-1}	2C (~90 mAh g^{-1})
O3-$Na_{0.8}Ni_{0.4}Ti_{0.6}O_2$ ref. 40	250 cycles (75%)	~83 mAh g^{-1}	1C (~63 mAh g^{-1})
O3-$NaNi_{0.4}Fe_{0.2}Mn_{0.2}Ti_{0.2}O_2$ ref. 47	200 cycles (84%)	~145mAh g^{-1}	2C (~75 mAh g^{-1})
O3-$Na[NiCoFeTi]_{1/4}O_2$ ref. 48	400 cycles (75%)	~116 mAh g^{-1}	5C (~102 mAh g^{-1})
P2-$Na_{2/3}MnO_2$ ref. 23	25 cycles (77%)	~175 mAh g^{-1}	––
P2-$Na_{2/3}Fe_{1/2}Mn_{1/2}O_2$ ref.12	30 cycles (82%)	~190 mAh g^{-1}	4C (~65 mAh g^{-1})
P2/O3-$Na_{0.66}Li_{0.18}Mn_{0.71}Ni_{0.21}Co_{0.08}O_2$ ref. 46	150 cycles (75%)	~200 mAh g^{-1}	5C (~69 mAh g^{-1})
P2-$Na_{0.80}Li_{0.12}Ni_{0.22}Mn_{0.66}O_2$ ref. 49	50 cycles (~91%)	~119 mAh g^{-1}	5C (~71 mAh g^{-1})
